# Assessing the presence of shared genetic architecture between Alzheimer's disease and major depressive disorder using genome-wide association data

**DOI:** 10.1038/tp.2017.49

**Published:** 2017-04-18

**Authors:** J Gibson, T C Russ, M J Adams, T-K Clarke, D M Howard, L S Hall, A M Fernandez-Pujals, E M Wigmore, C Hayward, G Davies, A D Murray, B H Smith, D J Porteous, I J Deary, A M McIntosh

**Affiliations:** 1 Division of Psychiatry, Centre for Clinical Brain Sciences, University of Edinburgh, Edinburgh, UK; 2 Centre for Dementia Prevention, University of Edinburgh, Edinburgh, UK; 3 Alzheimer Scotland Dementia Research Centre, University of Edinburgh, Edinburgh, UK; 4 Centre for Cognitive Ageing & Cognitive Epidemiology, University of Edinburgh, Edinburgh, UK; 5 Institute for Genetics and Molecular Medicine, Western General Hospital, Edinburgh, UK; 6 Department of Psychology, University of Edinburgh, Edinburgh, UK; 7 Aberdeen Biomedical Imaging Centre, College of Life Sciences and Medicine, University of Aberdeen, Aberdeen, UK; 8 Division of Population Health Sciences, University of Dundee, Dundee, UK

## Abstract

Major depressive disorder (MDD) and Alzheimer's disease (AD) are both common in older age and frequently co-occur. Numerous phenotypic studies based on clinical diagnoses suggest that a history of depression increases risk of subsequent AD, although the basis of this relationship is uncertain. Both illnesses are polygenic, and shared genetic risk factors could explain some of the observed association. We used genotype data to test whether MDD and AD have an overlapping polygenic architecture in two large population-based cohorts, Generation Scotland's Scottish Family Health Study (GS:SFHS; *N*=19 889) and UK Biobank (*N*=25 118), and whether age of depression onset influences any relationship. Using two complementary techniques, we found no evidence that the disorders are influenced by common genetic variants. Using linkage disequilibrium score regression with genome-wide association study (GWAS) summary statistics from the International Genomics of Alzheimer's Project, we report no significant genetic correlation between AD and MDD (*r*_G_=−0.103, *P*=0.59). Polygenic risk scores (PRS) generated using summary data from International Genomics of Alzheimer's Project (IGAP) and the Psychiatric Genomics Consortium were used to assess potential pleiotropy between the disorders. PRS for MDD were nominally associated with participant-recalled AD family history in GS:SFHS, although this association did not survive multiple comparison testing. AD PRS were not associated with depression status or late-onset depression, and a survival analysis showed no association between age of depression onset and genetic risk for AD. This study found no evidence to support a common polygenic structure for AD and MDD, suggesting that the comorbidity of these disorders is not explained by common genetic variants.

## Introduction

Major depressive disorder (MDD) and Alzheimer's disease (AD) are among the leading causes of disability worldwide, affecting an estimated 350 million and 44 million people respectively.^1, 2^ They are both common conditions in older age and are frequently comorbid;^3^ however, their inter-relationship is complex and not well understood. Depression may be a risk factor for dementia, or part of the dementia prodrome,^4, 5^ even when preceding dementia onset by over 10 years.^6, 7^ Prior depression has been found to be associated with increased risk of AD,^8, 9^ with depressed patients 1.5 times as likely to develop AD.^10^ However, while this association has been replicated in some studies,^9, 11, 12^ others find no association,^13, 14^ or an association only in selected subgroups, such as late-life depression.^15, 16, 17^

Both AD and MDD are influenced by genetic factors,^18, 19^ however few studies have examined the genetic overlap between these illnesses. Candidate gene and genome-wide association studies (GWAS) of AD have identified a number of variants associated with risk of developing late-onset AD, which accounts for over 95% of AD cases.^20, 21^ Common single-nucleotide polymorphisms (SNPs) explain 33% of the total phenotypic variance, with the strongest genetic risk factor being the APOE ɛ4 allele.^22^ MDD is a complex and phenotypically heterogeneous disorder, influenced by both genetic and environmental factors. GWAS of MDD have had limited success in identifying individual causal variants, probably due to underpowered samples and disease heterogeneity; however, two studies have identified some depression risk loci exceeding genome-wide significance.^23, 24^ Depression has a polygenic pattern of inheritance, with common variants estimated to explain 21% of the total phenotypic variance.^25^

Shared genetic risk factors could explain some of the observed association between these disorders. A number of studies have suggested that the known AD risk genes APOE and CR1 are also associated with MDD,^26, 27, 28, 29^ although these were candidate gene studies with small sample sizes, and the findings were inconsistent.^30, 31^ However, both disorders are highly polygenic,^18, 19^ and any genetic overlap could extend well beyond a few individual genes, although there is little evidence of this to date. A recent study estimating genetic correlations between a number of common disorders found no overlap in the genes associated with MDD and AD.^32^ An analysis investigating differences in shared genetic risk between early- and later-onset MDD with frequently comorbid conditions also found no association between these disorders.^33^ Here we sought to expand on this work, investigating potential polygenic associations in two large population cohorts, considering whether genetic risk for depression is associated with having a family history of Alzheimer's disease, and focusing on whether age of depression onset influences any relationship.

This study uses GWAS summary data and two complementary techniques, linkage disequilibrium (LD) score regression and polygenic profile scoring, to assess whether MDD and AD have an overlapping polygenic architecture in two large, independent UK-based population cohorts. These techniques are reliant on different metrics for assessing pleiotropy between traits, although both depend on the analyzed trait being polygenic in nature, with many genetic variants of individually small effect contributing to the overall phenotypic variation.

LD score regression^32, 34^ involves regressing summary statistics from many genetic variants onto their ‘LD score'—a measure of each variant's ability to tag local variants. For genetically influenced traits, variants with higher LD scores are more likely to tag causal variants and thus have higher test statistics on average than variants with low LD scores. This can be distinguished from inflation of test statistics due to population stratification and cryptic relatedness, as inflation resulting from LD drops off in a predictable manner as LD decreases, whereas that due to confounding does not. Thus the level of polygenicity between traits can be quantified, and the size and significance of genetic correlation estimated.

To test the extent to which shared genetic architecture is predictive of phenotypic variation in the samples on an individual subject basis, GWAS summary data can be used to calculate polygenic risk scores (PRS) for the traits under investigation. Association test statistics for each SNP in a ‘training' data set are used to weight alleles according to their association with disease risk, then these weightings are applied to genotypes in an independent data set to compute aggregate genomic PRS for each individual.^35^ Scores are assessed for their association with case versus control status, and PRS predictive of the trait can then be compared against observed phenotypes in individuals from that sample.

A high genetic correlation between MDD and AD, or a significant association between PRS for one disorder and case–control status of the other, would provide evidence that the two disorders may be influenced by overlapping genetic factors.

## Materials and methods

### Sample descriptions

This study used data from two large UK cohorts not previously utilized in large-scale consortia studies of MDD and AD: Generation Scotland's Scottish Family Health Study (GS:SFHS) and UK Biobank (UKB). These studies were approved by the relevant research ethics committees; the NHS Tayside Committee on Medical Research Ethics (Reference 05/S1401/89) for GS:SFHS, and the North West Multicentre Research Ethics Committee (Reference 11/NW/0382) for UKB. All individuals provided written informed consent.

#### Generation Scotland: Scottish Family Health Study

GS:SFHS is a family- and population-based cohort recruited through general medical practices across Scotland; the recruitment protocol and sample characteristics are described elsewhere.^36, 37^ In brief, the cohort consists of 23 960 individuals aged between 18 and 98 years, recruited if they had at least one other family member willing to participate. Pedigree information was available for all participants, detailed sociodemographic and clinical data were collected, and biological samples were taken for DNA extraction and genotyping. MDD status was determined by a screening questionnaire followed by the Structured Clinical Interview for the Diagnostic and Statistical Manual of Mental Disorders (SCID)^38^ for those who screened positive. Individuals diagnosed with bipolar disorder were excluded from this analysis.

#### UK Biobank

UKB is a health research resource, the sample characteristics of which are described elsewhere.^39, 40^ Briefly, this cohort consists of more than 500 000 individuals aged between 40 and 69 years, recruited from across the UK. Detailed sociodemographic data were collected, extensive baseline medical assessments carried out, and biological samples were taken for DNA extraction and genotyping. The depression phenotype was based on the putative definition of MDD established by Smith *et al.*,^41^ using responses to a touchscreen questionnaire, self-reported information, and linked electronic health records. Exclusions were made based on potentially confounding psychiatric disorders, related individuals in the sample, and inclusion in the GS:SFHS sample.

Further information on sample collection, DNA extraction, genotyping, quality control, and assessment of depression phenotype, for both samples, are provided in the Supplementary Materials.

### Polygenic risk scores for AD and MDD profiling

PRS for MDD and AD were created for all individuals with genotype data, incorporating all autosomal SNPs which passed quality control (see Supplementary Information for quality control parameters). PRS for AD were estimated using summary statistics from an independent GWAS of AD (17 008 AD cases, 37 154 controls), conducted by the International Genomics of Alzheimer's Project (IGAP).^18^ PRS for MDD were created using summary statistics from an independent GWAS data set from the Major Depressive Disorder working group of the Psychiatric Genomics Consortium (9240 MDD cases, 9519 controls).^19^ PRS were estimated using the PRSice software package, according to previously described protocols,^42^ with LD threshold and distance threshold for clumping of 0.2 and 300 kb respectively. Five scores were created for each individual, using SNPs selected according to the significance of their association with the phenotype in the discovery GWAS, at nominal *P*-value thresholds of 0.01, 0.05, 0.1, 0.5 and 1.0 (all SNPs).

Any individuals overlapping with the Psychiatric Genomics Consortium MDD GWAS sample were excluded from the study samples. Only GWAS summary data, not full genotypes, were available for the IGAP sample, thus we were unable to exclude the possibility of overlap between this sample and our study samples. However, as most IGAP participants were not from the UK,^18^ any overlap is likely to be very small.

### Statistical analysis

Two separate techniques were used to look for evidence of shared genetic architecture: LD score regression; and polygenic risk score analysis. PRS were also used to assess whether genetic risk for AD is associated with depression age of onset (AOO), or with ‘early-onset' or ‘late-onset' depression, via survival analyses and sub-setting the data by AOO.

Power calculations were implemented in AVENGEME, following the method outlined by Palla and Dudbridge.^43, 44^ The proportions of trait variance explained by SNPs on common GWAS arrays were taken from published sources, 0.21 for MDD^25^ and 0.33 for AD.^22^ We assumed an additive genetic covariance between the MDD training and target samples of 0.15 (likely a conservative estimate, given Palla and Dudbridge's covariance estimates for MDD-bipolar disorder and MDD-schizophrenia of 0.13 and 0.17, respectively^44^), and between MDD and AD of 0.05. The proportion of null markers was estimated by summing the excess of SNPs with lower than expected *P*-values under the assumption that *P*-values have a uniform distribution.

#### Linkage disequilibrium score regression

Cross-trait LD score regression was used to assess for any overlap in genetic architecture between MDD and AD. The method was applied to data from GWAS analyses of MDD in the study samples (GS:SFHS: *N*=19 809, 2648 cases, sample prevalence 13.4%, UKB: *N*=24 048, 8152 cases, sample prevalence 33.9%), and summary statistics from the AD GWAS by IGAP (sample prevalence 31.4%). As there were only 26 self-reported AD cases in GS:SFHS, and five cases in UKB, based on a combination of self-reported data and ICD diagnoses, we were unable to perform GWAS of AD in these samples. We followed the protocol outlined by Bulik-Sullivan *et al.*;^34^ full details of the methodology are provided in the Supplementary Materials. Following diagnostic checks, the GS:SFHS MDD data were found to have insufficient evidence of a clear polygenic signal and was excluded from further LD score regression analysis. LD score output was converted from the observed scale to the liability scale by supplying sample and population prevalence estimates for each trait (taken as 1.3% for AD^45^ and 19% for depression^46^); the different MDD prevalence estimates in the GS:SFHS and UKB samples did not substantially affect the correlation estimates.

#### Polygenic risk score analysis

PRS for MDD were assessed for their association with depression case–control status. Corresponding analysis was not possible for AD as the study sample age ranges did not include the demographic group where AD is most prevalent, resulting in too few cases. However, a proxy AD status was determined based on having a self-reported positive family history of AD (father, mother, sibling or grandparent for GS:SFHS, *N*=3116; father, mother or sibling for UKB, *N*=4149). After testing the prediction accuracy of AD PRS with the AD family history variable in the full samples, this analysis was repeated on a sub-sample of unrelated GS:SFHS participants, to ensure results were not confounded by family structure. We then tested for associations between polygenic risk for AD and MDD status, and between MDD PRS and AD family history. To ensure that any genetic correlation between AD and MDD PRS was not driven by a single locus, the analysis was repeated using MDD PRS recalculated with a 1000 kb region centered on the APOE locus removed.

For GS:SFHS, generalized linear mixed models were implemented in the ASReml-R (version 3.2.1) software package (www.vsni.co.uk/software/asreml) in R,^47^ with MDD or AD family history status as the dependent variable and PRS for MDD or AD, scaled to have a mean of 0 and standard deviation of 1, fitted as the predictor variable. Sex, age, age^2^ and the first four principal components from an ancestry-informative PC analysis were fitted as covariates in all GS:SFHS analyses. As this cohort is family-based, relatedness in the sample was controlled for by using pedigree kinship information to fit an additive genetic relationship matrix as a random effect. The dependent variable was a binary disease status, so a Taylor series approximation was used to transform the fixed effects and standard errors from the linear scale to the liability scale, according to previously described methods.^48^ The significance of the PRS in predicting disease outcome was estimated by the Wald F-statistic, conditional on the other fixed and random effects. Significant effects are reported where they survived Bonferroni correction for multiple testing, applied over the five thresholds.

The UKB sample consisted of unrelated individuals, so logistic regression was performed using generalized linear models in R, using a logit link function to account for the binary response variable. Fifteen principal components from a PC analysis, sex, age and age^2^ were fitted as covariates for all UKB analyses.

#### Age of depression onset

If late-life depression is an early indicator of dementia, then genetic risk for AD could be related to age of depression onset, such that individuals who become depressed later in life have higher genetic risk of developing AD. We tested this theory via two different approaches: MDD survival analysis and sub-setting the data by depression AOO. While almost all MDD cases in GS:SFHS (97.2%) include AOO data, equivalent data for the UKB sample was largely unavailable, with only a small proportion (22.7%) of MDD cases providing self-reported age of diagnosis information. In addition, the mean diagnosis age for UKB was 42 years, significantly older than the typically reported mean AOO for depression;^49^ this is likely due to the amount of missing data. Despite the shortcomings of this variable, we decided to proceed with the age of onset analysis in this sample, to replicate any findings in the better-informed GS:SFHS analysis, but keeping these limitations in mind when interpreting results.

We first conducted survival analyses using right censored data in Cox proportional hazards models. MDD cases had age of depression onset (GS:SFHS) or diagnosis (UKB) as the event time, while individuals without depression had age at assessment as the censoring time. For GS:SFHS, the unrelated sample was used in the survival analysis; 6932 individuals were included, with 961 events. For UKB, the sample size was 18 063, with 2075 events.

We then investigated whether differences in shared genetic risk exist between early- and later-onset MDD with AD. AD PRS were tested for association with early-onset and late-onset MDD, where early- and late-onset were defined firstly by using the first and last quartiles of AOO, and secondly using subsets of MDD cases experiencing their first depressive episode before or after the age of 40 years. While not ideal for isolating ‘late-onset' cases, this age threshold has previously been used,^50, 51^ and is certainly above the typical onset age for depression.^49^ In this instance, it also maximized the number of late-onset cases; using a later cutoff, for example, over 60 years, while more likely to reflect true late-onset depression, reduced the number of late-onset cases (GS:SFHS *N*=22, UKB *N*=132) below that required for adequately powered analysis. The late-onset analysis was restricted to only participants over 40 years old.

## Results

For the current study, the GS:SFHS sample consisted of 19 889 individuals (58.9% female, mean age 47.4 years, s.d.=15.0) for whom both phenotype and genetic data were available. Of these, 2 654 (13.3%) had a lifetime diagnosis of MDD, with mean (s.d.) age of onset 32.0 (13.0) years, and 17 235 (86.7%) were controls. The UKB sample used here consisted of 25 118 individuals (50.3% female, mean age 57.2 years, s.d.=7.9) with the relevant phenotype and genetic data. Of these, 9130 (36.4%) were assigned case status and 15 988 (63.6%) were designated as controls. The high prevalence of MDD in this sample is due to the exclusion of large numbers of controls, owing to incomplete and the missing data, and a lower threshold for case definition.^41^ Two thousand sevent five of the MDD cases had the data on self-reported age of depression diagnosis, with the mean (s.d.) diagnosis age being 42.1 (13.2) years.

### LD score regression

Diagnostic tests on the AD summary statistics from IGAP, and MDD GWAS summary statistics from the two study samples gave mean *χ*^2^ estimates of 1.114 for the AD data and 1.046 for UKB MDD data, indicating suitability for carrying out LD score regression. However, a mean *χ*^2^ of 1.015 for GS:SFHS MDD data indicated that these data were not suitable for this technique, owing to limited polygenic signal for MDD in the sample. The estimate of genetic correlation between the IGAP AD data and UKB MDD data was not significantly different from zero (*r*_G_=−0.103 (0.190), *Z*-score=−0.540, *P*-value=0.589), indicating little overlap in their genetic architecture.

### Polygenic risk score analysis

MDD PRS were positively associated with lifetime history of MDD at all P-value thresholds in both samples; this association was statistically significant at all thresholds in UKB, and four out of five thresholds in GS:SFHS (Supplementary Table 1). All significant results survived Bonferroni correction for multiple testing. The non-significant association at the *P*⩽0.01 threshold in GS:SFHS may reflect the reduced power at this threshold (41% power using the parameter estimates outlined in the methods, compared to 76–98% power for the other *P*-value thresholds). The larger UKB sample, with a higher prevalence of depression, had over 90% power at all thresholds. Individuals carrying more MDD risk alleles were significantly more likely to have a lifetime MDD diagnosis. The greatest proportion of variance explained (*r*^2^) was using a P-value threshold of *P*⩽1.0 (all SNPs) for both samples, although in both cases this was less than 1% (GS:SFHS: zβ=0.086 (0.045, 0.128), *P*=5.24 × 10^−5^, *r*^2^=8.59 × 10^−4^, UKB: zβ=0.073 (0.046, 0.100), *P*=1.01 × 10^−7^, *r*^2^=8.92 × 10^−4^).

AD PRS showed a statistically significant positive association with AD family history at all *P*-value thresholds in GS:SFHS (both full sample and the subset of unrelated individuals) and UKB, with all results surviving multiple testing correction (Supplementary Table 2). Individuals carrying more AD risk alleles were more likely to have a family member with AD. The greatest amount of variance explained was using a *P*-value threshold of *P*⩽0.01 (GS:SFHS full sample: zβ=0.078 (0.041, 0.116), *P*=4.38 × 10^−5^, *r*^2^=1.27 × 10^−3^, UKB: zβ=0.118 (0.084, 0.052), *P*=7.03 × 10^−12^, *r*^2^=2.15 × 10^−3^). However, we were unable to be certain that the IGAP training sample and the two study samples were completely independent, so the possibility remains that this effect may be inflated if there is sample overlap.

PRS for MDD were positively associated with AD family history in GS:SFHS; the association was nominally significant at three *P*-value thresholds, although these did not survive multiple testing correction (Table 1). The strongest association was found using a *P*-value threshold of *P*⩽0.05, (zβ=0.048 (0.009, 0.086), *P*=1.55 × 10^−2^). Repeating this analysis using MDD PRS with the APOE region excluded produced similar results (Supplementary Table 3). However, this association was not observed in UKB, where MDD PRS were not associated at even nominal levels with AD family history (Table 1).

Polygenic risk for AD was not associated with MDD status at even nominal levels of significance at any *P*-value threshold in either sample (Table 2).

### Age of depression onset analysis

Survival analysis using Cox proportional hazards models indicated that PRS for AD were not associated with age of depression onset/diagnosis at any *P*-value threshold in either study sample (Table 3), suggesting that genetic risk for AD does not influence the time to development of depression.

Sub-setting the data to consider associations between AD PRS and early-onset or late-onset depression, where 'early-onset' was defined as either the first quartile of AOO (GS:SFHS: 585 cases, 17 235 controls, UKB: 508 cases, 15 988 controls) or as depression onset under the age of 40 years (GS:SFHS: 1933 cases, 17 235 controls, UKB: 817 cases, 15 988 controls), and 'late-onset' was defined as either the fourth quartile of AOO (GS:SFHS: 637 cases, 11 561 controls, UKB: 508 cases, 12 135 controls) or as depression onset over the age of 40 years (GS:SFHS: 637 cases, 11 561 controls, UKB: 1215 cases, 15 794 controls) confirmed that neither early-onset nor late-onset depression were associated with AD PRS in these samples. There were no significant associations at any *P*-value threshold for either the late-onset or early-onset depression subgroups in either sample (Table 4).

Taking into account the number of LD pruned (and thus assumed independent) SNPs in common between the IGAP AD GWAS and our GS:SFHS and UKB samples, (134 152 and 227 830 respectively), and the assumptions outlined in the methods, this analysis had sufficient power to detect associations between AD PRS and depression status when the full data sets were used (>80% for GS:SFHS, >90% for UKB). However, the power was considerably reduced in the AOO analysis due to smaller sample sizes and lower disease prevalence after sub-setting. The power for these analyses ranged between 16 and 73% (Supplementary Table 4).

## Discussion

This study provides little evidence for overlap in the polygenic architecture of lifetime MDD and AD, based on either cross-trait LD score regression or a polygenic profile score approach in two large, independent population cohorts. The low and non-significant point estimate of genetic correlation indicates that the phenotypic correlations between the disorders are not largely influenced by common genetic variants. PRS for AD were not associated with lifetime history of MDD, late-onset MDD, or age of depression onset. PRS for MDD were positively associated with a family history of AD in GS:SFHS, but this association was not statistically significant and was not replicated in UKB.

There is considerable evidence for the comorbidity between AD and MDD, particularly late-onset depression, with many studies suggesting it may be a prodrome of AD.^17, 52, 53, 54^ Depressive symptoms are commonly found in AD, and a lifetime history of depression appears to increase risk of developing AD, with greater frequency and severity of depressive symptoms further increasing this risk.^55, 56^ However, causality is difficult to establish; the relationship remains controversial and findings are inconsistent—perhaps not surprising given the heterogeneity of both disorders, and the arbitrary threshold criteria applied to clinical diagnosis, especially for depression.^57^

Phenotypic correlations between the disorders have been extensively researched, but far fewer studies have assessed the potential contribution of shared genetics. While some previous studies have found associations between the single, large-effect AD risk genes APOE and CR1, and depression,^26, 27, 28, 29^ other studies failed to report this;^30, 31^ these small-sample, primarily candidate gene studies do not provide convincing evidence. Here an aggregate score of all genetic variants associated with AD was not found to be associated with MDD, suggesting the genetic overlap is restricted to a few genes, or that AD risk genes only influence a subtype of depression which has not been isolated in this investigation.

This study did find a nominally significant association between polygenic risk for MDD and a family history of AD in GS:SFHS, although this was not replicated in UKB, nor validated by LD score regression. This anomalous result does not provide compelling support for a shared genetic architecture; the association failed to exceed statistical significance, and the AD family history variable may also reflect non-genetic familial factors and is potentially inaccurate, confounded by mood state and subject to recall bias.

In contrast to other psychiatric disorders, GWAS of MDD have had limited success; despite evidence supporting its heritability, it has proven difficult to establish associated genetic variants.^19^ The scarcity of replicable causal variants detected by GWAS is probably attributable to two main factors: underpowered sample sizes and disease heterogeneity.^58, 59, 60, 61, 62^ Simulations suggest that depression GWAS will need sample sizes up to five times larger than those of schizophrenia or bipolar disorder to have comparable power, owing to the disorder's higher prevalence, lower heritability, and the smaller effect sizes involved.^19, 63^ However, increasingly large samples and approaches to account for the multiple subtypes of depression will increase the power of genetic studies. Two recently published studies have revealed the first identified depression risk loci: a GWAS of MDD in a phenotypically homogeneous sample of female Han Chinese identified two loci exceeding genome-wide significance,^23^ and a meta-analysis combining 23andMe and PGC MDD GWAS data identified 17 significantly associated SNPs.^24^

MDD is hugely heterogeneous—it is possible to meet DSM diagnostic criteria for major depression through at least 227 different symptom combinations, some of which are opposites.^64^ Aggregating these biologically different subtypes into a single category could be contributing to the limited success of genetic studies. If subtypes associate with differing pathophysiological correlates, this could indicate that they may also have partially distinct genetic liabilities. A recent study provides evidence of different polygenic signatures for MDD subtypes: dissecting MDD along typical and atypical symptom profiles, typical depression was strongly associated with schizophrenia PRS, while atypical depression was associated with PRS for body mass index and triglycerides.^65^

Age of depression onset is another well-documented source of phenotypic variation, and early- and late-onset depression could represent distinct subgroups. Different characteristics have been reported between patients with varying AOO: increased familial loading for MDD amongst patients with childhood depression, higher prevalence of comorbid personality disorders and neuroticism characterising early-adult onset, later adult onset associated with environmental risk factors, and geriatric depression associated with more vascular risk factors.^66, 67^ Literature regarding how depression AOO influences risk of developing AD has been inconsistent. Some studies suggest that even depression occurring many years before dementia onset increases risk,^6, 7^ while others found that only late-life depression is associated with dementia.^17^ If MDD subtypes representing more homogeneous phenotypes are characterized by partially distinct genetic liabilities, the genetic basis of early- and late-onset depression may differ. Earlier-onset depression has been reported to have greater genetic overlap with schizophrenia and bipolar disorder than late-onset depression, suggesting that genetic susceptibility to MDD may indeed differ with age of disease onset.^33^ In contrast, this study, using AD PRS in a proportional hazards model, and assessing the influence of AD PRS on both early- and later-onset depression, reports no association between genetic risk of AD and depression AOO, despite the well-documented comorbidity with late-onset MDD. This result is consistent with the above-mentioned study, which also found no association between MDD status and genetic risk for AD, regardless of depression AOO.^33^

The main findings of this study suggest that the genetic basis of the two disorders are largely distinct. This is consistent with the small number of other studies using genotype data reported to date,^32, 33^ and extends previous work to consider the influence of MDD genetic risk on AD status, using family history of AD as proxy, and further investigating the effect of age of depression onset. Major strengths of this study include the use of two different methods of detecting pleiotropy, in two large, independent population samples, with results likely generalizable at least to UK and other northern European populations.

This investigation has a number of limitations. A power analysis using AVENGEME and based on plausible figures extracted from the literature suggests that the study is adequately powered when the full samples are used. However, having completed the analysis, our effect sizes are very small, indicating that either we are correct in not rejecting a null hypothesis of no genetic association between the disorders, or that the study is underpowered to detect a significant effect of the size found here. The AOO analysis, using subsets of cases with depression onset in specific age ranges, is underpowered to detect associations, so the results cannot be considered conclusive.

Arguably, the disparity in ascertainment of MDD status between samples could be a limitation, although the consistency of findings in spite of this is reassuring, indicating that the results are robust to the diagnosis method used.

Using binary measures of depression may limit our power to detect any association,^68^ as there is evidence from several longitudinal studies that number of depressive symptoms at baseline predicts development of AD with an approximately linear relationship.^9, 10^ If even mild symptoms increase risk of developing AD, defining MDD as a binary trait may underestimate any association. The GWAS data used for this study simply examined ‘depressed' cases versus controls, potentially combining multiple disorder subtypes that vary in their genetic etiology, and undermining the power of the genetic analyses to detect associations.

The participants in the study samples, with mean ages of 47 and 57 years in GS:SFHS and UKB, respectively, were largely too young to express clinical symptoms of AD. We were therefore unable to carry out GWAS for AD in either sample, or to test whether the estimated PRS for AD accurately predicted disease outcome. However, the proxy AD family history variable was associated with AD PRS, indicating that in genetic analyses family history of AD can act as an acceptable substitute in non-clinical samples. A recent study by Liu *et al.*^69^ using family history of a disease as a phenotype to increase power to detect association further demonstrates the validity of this approach.

While the survival analysis and AOO analyses carried out here found no evidence for association between genetic risk for AD and age of depression onset, the age ranges of sample participants meant that there are unlikely to be many individuals with true 'late-onset' depression which could precede dementia onset in AD. Depression AOO has been suggested to have a bimodal distribution, with most first episodes occurring around the age of 20 years, and a smaller, late-onset peak around 70–80 years.^70^ The samples studied may be too young to determine whether an age-dependent effect exists. In addition, both the quality and quantity of depression AOO data for the UKB sample are poor. Nonetheless, the UKB AOO results closely match those found for the GS:SFHS sample, where the AOO data were more reliable.

Given the limited power of the AOO analysis, and the training GWAS data defining lifetime depression only, the largely null results of this investigation do not preclude the possibility of a genetic association between Alzheimer's disease and a well-defined subgroup of depression cases characterized by late-life onset. Many studies indicate that late-life depression may be a prodromal feature of AD, and it remains possible that a genetic correlation exists between these two phenotypes, but the effect is not detected in this or other analyses using lifetime depression due to dilution.

The basis of the frequently observed comorbidity of MDD and AD remains uncertain, however this investigation suggests that it is not largely driven by genetic factors, at least when considering lifetime, rather than specifically late-onset MDD. A number of other hypotheses are possible, including common environmental or epigenetic risk factors,^71, 72, 73^ depression compromising cognitive reserve,^4^ insight of cognitive decline causing depressive symptoms,^4, 14^ or depression contributing directly to cognitive decline, perhaps via damage to neural systems.^4, 74, 75^ These hypotheses are not mutually exclusive, and multiple types of interaction are likely to be involved.

It is clear that further research is needed to understand the biological mechanisms that account for the complex relationship between these disorders, including consideration of common environmental factors or other non-genetic causes. Elucidating the nature of this relationship could enable development of novel interventions to reduce the considerable burden on those affected, and allow better clinical outcomes.

## Figures and Tables

**Table 1:** Associations between polygenic risk scores for MDD and family history of AD at five different *P*-value thresholds in the GS:SFHS and UKB samples

*MDD PGRS* P*-value threshold*	*GS:SFHS*			*UKB*		
	*Beta*	*95% CI*	P*-value*^a^	*Beta*	*95% CI*	P*-value*^a^
⩽0.01	0.023	−0.016, 0.062	2.55 × 10^−1^	0.003	−0.031, 0.036	8.83 × 10^−1^
⩽0.05	0.048	0.009, 0.086	1.55 × 10^−2^	0.000	−0.034, 0.034	9.86 × 10^−1^
⩽0.10	0.037	−0.001, 0.076	6.04 × 10^−2^	0.010	−0.024, 0.044	5.59 × 10^−1^
⩽0.50	0.040	0.002, 0.079	4.17 × 10^−2^	0.017	−0.017, 0.051	3.30 × 10^−1^
⩽1.00	0.039	0.000, 0.077	4.99 × 10^−2^	0.016	−0.019, 0.050	3.66 × 10^−1^

Abbreviations: AD, Alzheimer's disease; CI, confidence interval; GS:SFHS, Generation Scotland's Scottish Family Health Study; MDD, major depressive disorder; PGRS, polygenic risk scores; UKB, UK Biobank.

a *P*-values shown are uncorrected for multiple testing. None of the significant *P*-values shown survived Bonferroni correction for multiple testing.

**Table 2:** Associations between polygenic risk scores for Alzheimer's disease and depression status at five different P-value thresholds in the GS:SFHS and UKB samples

*AD PGRS* P*-value threshold*	*GS:SFHS*			*UKB*		
	*Beta*	*95% CI*	P*-value*^a^	*Beta*	*95% CI*	P*-value*^a^
⩽0.01	−0.012	−0.056, 0.031	5.84 × 10^−1^	0.025	−0.002, 0.051	6.64 × 10^−2^
⩽0.05	0.006	−0.037, 0.049	7.73 × 10^−1^	0.015	−0.012, 0.041	2.77 × 10^−1^
⩽0.10	0.009	−0.034, 0.052	6.92 × 10^−1^	0.022	−0.004, 0.049	9.60 × 10^−2^
⩽0.50	−0.010	−0.053, 0.033	6.62 × 10^−1^	0.011	−0.015, 0.038	3.96 × 10^−1^
⩽1.00	−0.011	−0.054, 0.032	6.30 × 10^−1^	0.009	−0.017, 0.036	4.86 × 10^−1^

Abbreviations: AD, Alzheimer's disease; CI, confidence interval; GS:SFHS, Generation Scotland's Scottish Family Health Study; PGRS, polygenic risk scores; UKB, UK Biobank.

a *P*-values shown are uncorrected for multiple testing.

**Table 3:** Survival analysis for age of depression onset (GS:SFHS) or diagnosis (UKB) and polygenic risk for Alzheimer's disease, from Cox proportional hazards model. Results are shown at five different *P*-value thresholds

*AD PGRS P-value threshold*	*GS:SFHS*			*UKB*		
	*Hazard ratio*	*95% CI*	P*-value*^a^	*Hazard ratio*	*95% CI*	P*-value*^a^
⩽0.01	0.999	0.938, 1.063	9.75 × 10^−1^	1.014	0.972, 1.058	5.23 × 10^−1^
⩽0.05	0.992	0.931, 1.056	7.91 × 10^−1^	0.984	0.943, 1.028	4.73 × 10^−1^
⩽0.10	0.986	0.926, 1.049	6.49 × 10^−1^	0.994	0.952, 1.037	7.70 × 10^−1^
⩽0.50	0.976	0.917, 1.038	4.39 × 10^−1^	0.988	0.947, 1.032	5.91 × 10^−1^
⩽1.00	0.969	0.910, 1.031	3.15 × 10^−1^	0.986	0.945, 1.030	5.33 × 10^−1^

Abbreviations: AD, Alzheimer's disease; CI, confidence interval; GS:SFHS, Generation Scotland's Scottish Family Health Study; PGRS, polygenic risk scores; UKB, UK Biobank.

a *P*-values shown are uncorrected for multiple testing.

**Table 4:** Associations between polygenic risk scores for AD and early-onset depression status, and AD PGRS and late-onset depression, at five different *P*-value thresholds in the GS:SFHS and UKB samples

*AD PGRS P-value threshold*	*GS:SFHS*			*UKB*		
	*Beta*	*95% CI*	P*-value*^a^	*Beta*	*95% CI*	P*-value*^a^
*First quartile of age of onset*						
⩽0.01	0.001	−0.023, 0.024	9.16 × 10^−1^	0.026	−0.063, 0.115	5.64 × 10^−1^
⩽0.05	0.001	−0.023, 0.024	9.38 × 10^−1^	0.019	−0.070, 0.109	6.73 × 10^−1^
⩽0.10	0.001	−0.023, 0.024	9.23 × 10^−1^	0.007	−0.083, 0.097	8.82 × 10^−1^
⩽0.50	−0.008	−0.032, 0.016	4.84 × 10^−1^	−0.021	−0.110, 0.069	6.54 × 10^−1^
⩽1.00	−0.007	−0.031, 0.017	5.40 × 10^−1^	−0.028	0.118, 0.062	5.37 × 10^−1^
						
*Fourth quartile of age of onset*						
⩽0.01	0.013	−0.022, 0.048	4.51 × 10^−1^	0.020	−0.069, 0.109	6.56 × 10^−1^
⩽0.05	0.016	−0.019, 0.051	3.78 × 10^−1^	−0.039	−0.128, 0.051	4.00 × 10^−1^
⩽0.10	0.008	−0.027, 0.043	6.49 × 10^−1^	−0.025	−0.114, 0.065	5.91 × 10^−1^
⩽0.50	−0.004	−0.039, 0.032	8.60 × 10^−1^	0.007	−0.082, 0.096	8.77 × 10^−1^
⩽1.00	−0.006	−0.041, 0.029	7.40 × 10^−1^	0.012	−0.077, 0.101	7.86 × 10^−1^
						
*MDD onset over 40 years*						
⩽0.01	0.013	−0.022, 0.048	4.51 × 10^−1^	0.002	−0.057, 0.061	9.53 × 10^−1^
⩽0.05	0.016	−0.019, 0.051	3.78 × 10^−1^	−0.032	−0.091, 0.027	2.91 × 10^−1^
⩽0.10	0.008	−0.027, 0.043	6.49 × 10^−1^	−0.013	−0.072, 0.046	6.62 × 10^−1^
⩽0.50	−0.004	−0.039, 0.032	8.60 × 10^−1^	−0.019	−0.078, 0.041	5.35 × 10^−1^
⩽1.00	−0.006	−0.041, 0.029	7.40 × 10^−1^	−0.015	−0.074, 0.044	6.13 × 10^−1^
						
*MDD onset under 41 years*						
⩽0.01	−0.020	−0.058, 0.019	3.20 × 10^−1^	0.0016	−0.056, 0.087	6.63 × 10^−1^
⩽0.05	0.001	−0.038, 0.039	9.55 × 10^−1^	−0.015	−0.087, 0.057	6.83 × 10^−1^
⩽0.10	0.007	−0.031, 0.045	7.30 × 10^−1^	−0.019	−0.091, 0.053	6.09 × 10^−1^
⩽0.50	−0.004	−0.043, 0.034	8.16 × 10^−1^	−0.031	−0.102, 0.041	4.06 × 10^−1^
⩽1.00	−0.004	−0.043, 0.034	8.25 × 10^−1^	−0.039	−0.111, 0.033	2.90 × 10^−1^

Abbreviations: AD, Alzheimer's disease; CI, confidence interval; GS:SFHS, Generation Scotland's Scottish Family Health Study; MDD, major depressive disorder; PGRS, polygenic risk scores; UKB, UK Biobank.

a *P*-values shown are uncorrected for multiple testing.
