# Supplementary material for: Assessing the presence of shared genetic architecture between Alzheimer's disease and major depressive disorder using genome-wide association data
Source: Transl Psychiatry. 2017 Apr 18;7(4):e1094–. doi: 10.1038/tp.2017.49 (PMC5416691; doi:10.1038/tp.2017.49)
Supplement: Supplementary Information [file tp201749x1.docx]

**This document contains the supplementary information for the manuscript by Gibson et al. “Assessing the presence of shared genetic architecture between Alzheimer’s disease and major depressive disorder using genome-wide association data”.**

**Supplementary Information**

*Genotyping and quality control in GS:SFHS*

Blood and saliva samples were collected, following standard operating procedures, and stored at the Wellcome Trust Clinical Research Facility Genetics Core, Edinburgh, UK ([www.wtcrf.ed.ac.uk](http://www.wtcrf.ed.ac.uk/)), where DNA extraction and genotyping were carried out; details of sample collection and DNA extraction are provided elsewhere^1^. Samples were genotyped using the Illumina HumanOmniExpressExome-8v1.0 Beadchip and Infinium chemistry^2^ and processed using the Illumina GenomeStudio Analysis software v2011.1 (Illumina, San Diego, CA, USA). Quality control procedures on the raw genotypes removed individuals with an overall genotyping rate of less than 99%, SNPs with a minor allele frequency less than 1% or call rate under 99%, or a significant deviation from Hardy-Weinberg equilibrium (P≥1x10^-6^).

*Ascertainment of MDD phenotype in GS:SFHS*

A screening questionnaire consisting of two questions (“Have you ever seen anybody for emotional or psychiatric problems?” and “Was there ever a time when you, or someone else, thought you should see someone because of the way you were acting or feeling?”) was administered to all GS:SFHS participants to assess for symptoms of MDD. Those answering yes to either question (21.7% screened positive) were asked to complete the Structured Clinical Interview for the Diagnostic and Statistical Manual of Mental Disorders (SCID)^3^, which provided information on lifetime history of depression, age of onset, and number of depressive episodes. Individuals were subsequently diagnosed with MDD if they fulfilled DSM-IV criteria^4^, while those who screened negative in the initial questionnaire or who screened positive but did not meet the criteria for MDD were diagnosed as being free from MDD and were assigned control status for this analysis. Any participants who refused to complete either the screening questionnaire or the SCID had their MDD status recorded as 'missing' and were excluded from this analysis. Individuals with a diagnosis of bipolar disorder were also excluded.

*Genotyping and quality control in UKB*

Biological samples collected from UKB participants were held at the UK Biobank facility in Stockport, UK prior to DNA extraction; details of the UKB sampling and DNA extraction procedures are provided elsewhere^5,6^. Samples were genotyped at the Affymetrix Research Services Laboratory in Santa Clara, California, USA, using either the custom-designed UK Biobank Axiom genotyping array from Affymetrix or the closely related UK BiLEVE array, described in detail elsewhere^7^, which has over 95% common marker content with the UK Biobank Axiom array, and processed on the Affymetrix GeneTitan© Multi-Channel Instrument. Genotypes were then called using the Affymetrix Power Tools software and the Affymetrix Best Practices Workflow^8^. Quality control was carried out by Affymetrix, the Wellcome Trust Centre for Human Genetics, and the University of Edinburgh, and included removal of participants based on missingness, relatedness, gender mismatch, non-British ancestry and other criteria; full details of genotyping and quality control are given elsewhere^7^.

*Ascertainment of MDD phenotype in UKB*

MDD and bipolar symptoms, along with a number of other psychiatric disorders and symptoms, were assessed in UKB participants via a touchscreen assessment^9^. No specific psychiatric interview was carried out, however criteria for mood disorders followed the structured diagnostic approach within the International Classification of Diseases (ICD–10)^10^ and the American Psychiatric Association's DSM-IV^4^. The depression phenotype used for this analysis was a single, dichotomised case/control variable based on the putative definition of MDD established by Smith et al.^11^, using responses to the touchscreen questionnaire, self-reported information, and inpatient records via linkage to electronic health records. A number of exclusion criteria were applied based on self-reported information and linked health records, to limit potentially confounding psychiatric disorders within the sample. Individuals with either a self-declared diagnosis or an ICD classification of Parkinson's disease, bipolar disorder, multiple personality disorder, or schizophrenia, or an ICD classification of autism or intellectual disability, or who had ever had a prescription for antipsychotic medication were excluded from both cases and controls. Additionally, individuals who had ever been diagnosed with MDD, a mood disorder, or an anxiety disorder, or who had ever been prescribed antidepressant or anxiolytic medication were excluded from the control group. Related individuals and any participants who were also in the GS:SFHS sample were also excluded from the UKB analysis.

*LD Score Regression Methodology*

We followed the protocol outlined by Bulik-Sullivan et al.^12^ for LD score regression. Diagnostic checks were performed to ensure that the GWAS data sets had sufficient evidence of a clear polygenic signal, with a threshold of >1.02 for the mean χ^2^ statistic. The GS:SFHS MDD data failed to exceed this threshold; this sample was therefore excluded from further LD score regression analysis. We used LD scores and weights pre-computed using 1000 Genomes European data^13^ and downloaded from the Broad Institute (<http://www.broadinstitute.org/~bulik/eur_ldscores/>) - this European reference panel is suitable for use with outbred populations of predominantly northern European Ancestry, including the GS:SFHS and UKB samples used in this study. To control for imputation quality, only those SNPs which are also found in HapMap3^14^ with a MAF of > 0.05 were included in the analysis. Sample size was assumed to be the same for all SNPs. Any SNPs listed in the summary statistics files that did not match those listed in the 1000 Genomes data used to estimate the LD scores were assumed to be mislabelled, and were removed. As the degree of sample overlap between the IGAP AD dataset and the MDD samples was unknown, we used an unconstrained intercept in the regression model. We converted the LD score output from the observed scale to the liability scale by supplying the sample and population prevalences for each trait; different sample prevalence estimates such as the MDD prevalence in the GS:SFHS and UKB samples affects the estimates of SNP heritability, but does not substantially affect the genetic correlation.

**References**

1 Smith BH, Campbell H, Blackwood D, Connell J, Connor M, Deary IJ *et al.* Generation Scotland: the Scottish Family Health Study; a new resource for researching genes and heritability. *BMC Medical Genetics* 2006; **7:** 1-9.

2 Gunderson KL. Whole-genome genotyping on bead arrays. *Methods Mol Biol* 2009; **529:** 197-213.

3 First MB, Gibbon M, Spitzer RL, Williams JBW. *Structured clinical interview for DSM-IV-TR Axis Disorders (Research Version)*. Biometrics Research. New York State Psychiatric Institute: New York, 2002.

4 American Psychiatric Association. 2013. *Diagnostic and Statistical Manual of Mental Disorders, fifth Edition*.

5 UK Biobank (2015) *Genotyping of 500,000 participants: Description of sample processing workflow and preparation of DNA for genotyping*. Retrieved from

<https://biobank.ctsu.ox.ac.uk/crystal/docs/genotyping_sample_workflow.pdf>

6 UK Biobank (2014) *DNA extraction at UK Biobank.* Retrieved from http://www.ukbiobank.ac.uk/wp-­‐*content/uploads/2014/04/DNA-­‐Extraction-­‐at-­‐UK-­‐Biobank-­‐October-­‐2014.pdf*

7 Wain LV, Shrine N, Miller S, Jackson VE, Ntalla I, Soler Artigas M *et al.* Novel insights into the genetics of smoking behaviour, lung function, and chronic obstructive pulmonary disease (UK BiLEVE): a genetic association study in UK Biobank. *Lancet Respir Med* 2015; **3**(10)**:** 769-781.

8 Affymetrix (2014) *Axiom® Genotyping Solution Data Analysis Guide.* Retrieved from

http://media.affymetrix.com/support/downloads/manuals/axiom_genotyping_solution_analysi*s_guide.pdf*

*9* UK Biobank (2011) *Touchscreen questionnaire.* Retrieved from  *http://www.ukbiobank.ac.uk/wp-content/uploads/2011/06/Touch_screen_questionnaire.pdf*

10 World Health Organization. 1992. The ICD-10 Classification of Mental and Behavioural Disorders. International Classification 10: 1-267.

11 Smith DJ, Nicholl BI, Cullen B, Martin D, Ul-Haq Z, Evans J *et al.* Prevalence and characteristics of probable major depression and bipolar disorder within UK biobank: cross-sectional study of 172,751 participants. *PLoS One* 2013; **8**(11)**:** e75362.

12 Bulik-Sullivan BK, Loh P-R, Finucane HK, Ripke S, Yang J, Consortium SWGotPG*, et al*. LD Score regression distinguishes confounding from polygenicity in genome-wide association studies. *Nature Genetics* 2015; **47:** 291-295.

13 1000 Genomes Project Consortium. An integrated map of genetic variation from 1,092 human genomes. *Nature* 2012; **491:** 56–65.

14 International HapMap 3 Consortium. Integrating common and rare genetic variation in diverse human populations. *Nature* 2010; **467:** 52–58.

**Supplementary Table 1. Associations between polygenic profile scores for MDD and depression status at five different P-value thresholds in the GS:SFHS and UKB samples**

| *MDD PGRS p-value threshold* | *GS:SFHS* | | | | | *UKB* | | | |
| --- | --- | --- | --- | --- | --- | --- | --- | --- | --- |
|  | *Beta* | *95% CI* | *P-value** | *r^2^* | *Beta* | | *95% CI* | *P-value** | *r^2^* |
| *P≤0.01* | 0.034 | [-0.009, 0.077] | 1.23x10^-1^ | 1.01x10^-4^ | 0.046 | | [0.020, 0.072] | **6.13x10^-4^** | 3.69x10^-4^ |
| *P≤0.05* | 0.067 | [0.025, 0.110] | **1.85x10^-3^** | 4.82x10^-4^ | 0.059 | | [0.033, 0.086] | **1.21x10^-5^** | 6.02x10^-4^ |
| *P≤0.10* | 0.076 | [0.034, 0.118] | **4.37x10^-4^** | 6.31x10^-4^ | 0.066 | | [0.039, 0.092] | **1.42x10^-6^** | 7.31x10^-4^ |
| *P≤0.50* | 0.082 | [0.040, 0.124] | **1.27x10^-4^** | 7.69x10^-4^ | 0.073 | | [0.046, 0.100] | **1.01x10^-7^** | 8.92x10^-4^ |
| *P≤1.00* | 0.086 | [0.045, 0.128] | **5.24x10^-5^** | 8.59x10^-4^ | 0.073 | | [0.046, 0.100] | **1.01x10^-7^** | 8.92x10^-4^ |

*P-values shown are uncorrected for multiple testing. All highlighted P-values withstood Bonferroni correction for multiple testing.

**Supplementary Table 2. Associations between polygenic profile scores for AD and family history of AD at five different P-value thresholds in the GS:SFHS full sample, the unrelated individuals in GS:SFHS, and the UKB sample**

| *AD PGRS P-value threshold* | *GS:SFHS full sample* | | | | *GS:SFHS unrelated sample* | | | | *UKB sample* | | | |
| --- | --- | --- | --- | --- | --- | --- | --- | --- | --- | --- | --- | --- |
|  | *Beta* | *95% CI* | *P-value** | *r^2^* | *Beta* | *95% CI* | *P-value** | *r^2^* | *Beta* | *95% CI* | *P-value** | *r^2^* |
| *P≤0.01* | 0.078 | [0.041, 0.116] | **4.38x10^-5^** | 1.27x10^-3^ | 0.123 | [0.061, 0.185] | **1.07x10^-4^** | 2.37x10^-3^ | 0.118 | [0.084,0.152] | **7.03x10^-12^** | 2.15x10^-3^ |
| *P≤0.05* | 0.070 | [0.033, 0.108] | **2.57x10^-4^** | 9.94x10^-4^ | 0.107 | [0.045, 0.170] | **7.43x10^-4^** | 1.79x10^-3^ | 0.068 | [0.034,0.102] | **8.92x10^-5^** | 7.02x10^-4^ |
| *P≤0.10* | 0.065 | [0.028, 0.103] | **6.62x10^-4^** | 8.51x10^-4^ | 0.092 | [0.030, 0.154] | **3.56x10^-3^** | 1.34x10^-3^ | 0.073 | [0.039,0.107] | **2.35x10^-5^** | 8.17x10^-4^ |
| *P≤0.50* | 0.062 | [0.024, 0.100] | **1.26x10^-3^** | 6.90x10^-4^ | 0.082 | [0.020, 0.144] | **9.66x10^-3^** | 1.05x10^-3^ | 0.071 | [0.037,0.105] | **4.13x10^-5^** | 7.69x10^-4^ |
| *P≤1.00* | 0.062 | [0.024, 0.100] | **1.34x10^-3^** | 6.75x10^-4^ | 0.082 | [0.020, 0.144] | **9.88x10^-3^** | 1.05x10^-3^ | 0.068 | [0.034,0.102] | **8.71x10^-5^** | 7.04x10^-4^ |

*P-values shown are uncorrected for multiple testing. All highlighted P-values withstood Bonferroni correction for multiple testing.

**Supplementary Table 3. Associations between polygenic profile scores for MDD excluding the APOE region, and family history of AD at five different P-value thresholds in the GS:SFHS sample**

| *MDD minus APOE PGRS P-value threshold* | *Beta* | *95% CI* | *P-value** | *r^2^* |
| --- | --- | --- | --- | --- |
| *P≤0.01* | 0.022 | [-0.017, 0.061] | 2.55x10^-1^ | 7.76x10^-5^ |
| *P≤0.05* | 0.048 | [0.009, 0.086] | 1.55x10^-2^ | 3.81x10^-4^ |
| *P≤0.10* | 0.037 | [-0.002, 0.075] | 6.06x10^-2^ | 1.56x10^-4^ |
| *P≤0.50* | 0.040 | [0.002, 0.079] | 4.04x10^-2^ | 2.35x10^-4^ |
| *P≤1.00* | 0.039 | [0.000, 0.077] | 4.79x10^-2^ | 2.24x10^-4^ |

*P-values shown are uncorrected for multiple testing. None of the significant P-values withstood Bonferroni correction for multiple testing.

**Supplementary Table 4. Power analyses**

| *GS:SFHS* | | | | *UKB* | | | |
| --- | --- | --- | --- | --- | --- | --- | --- |
| *Predictor variable* | *Response variable* | *PRS P-value threshold* | *Power* | *Predictor variable* | *Response variable* | *PRS P-value threshold* | *Power* |
| *MDD PRS* | *MDD status* | 0.01  0.05  0.1  0.5  1 | 0.408  0.760  0.880  0.983  0.986 | *MDD PRS* | *MDD status* | 0.01  0.05  0.1  0.5  1 | 0.998  0.999  0.999  1.000  1.000 |
| *AD PRS* | *MDD status* | 0.01  0.05  0.1  0.5  1 | 0.722  0.816  0.833  0.837  0.835 | *AD PRS* | *MDD status* | 0.01  0.05  0.1  0.5  1 | 0.910  0.955  0.962  0.963  0.962 |
| *AD PRS* | *Early onset (1^st^ quartile of AOO) MDD* | 0.01  0.05  0.1  0.5  1 | 0.248  0.300  0.312  0.314  0.313 | *AD PRS* | *Early onset (1^st^ quartile of AOO) MDD* | 0.01  0.05  0.1  0.5  1 | 0.161  0.187  0.193  0.194  0.193 |
| *AD PRS* | *Early onset (under 41 years) MDD* | 0.01  0.05  0.1  0.5  1 | 0.610  0.710  0.730  0.735  0.732 | *AD PRS* | *Early onset (under 41 years) MDD* | 0.01  0.05  0.1  0.5  1 | 0.227  0.268  0.277  0.279  0.278 |
| *AD PRS* | *Late onset (4^th^ quartile of AOO) MDD* | 0.01  0.05  0.1  0.5  1 | 0.261  0.316  0.329  0.332  0.330 | *AD PRS* | *Late onset (4^th^ quartile of AOO) MDD* | 0.01  0.05  0.1  0.5  1 | 0.160  0.186  0.192  0.193  0.192 |
| *AD PRS* | *Late onset (over 40 years) MDD* | 0.01  0.05  0.1  0.5  1 | 0.261  0.316  0.329  0.332  0.330 | *AD PRS* | *Late onset (over 40 years) MDD* | 0.01  0.05  0.1  0.5  1 | 0.308  0.365  0.377  0.380  0.379 |

Power calculations were carried out in AVENGEME, following the method described by Palla and Dudbridge^1,2^. Plausible estimates for the proportion of trait variance explained by SNPs on common GWAS arrays for the two disorders under investigation were extracted from the literature: 0.21 for MDD^3^, and 0.33 for AD^4^. An additive genetic covariance of 0.15 between the MDD training and target samples was assumed, based on similar figures for other psychiatric disorders^2^. A covariance of 0.05 was assumed between MDD and AD. The proportion of null markers was estimated by summing the excess of SNPs with lower than expected P-values under the assumption that P-values have a uniform distribution: the values were 0.8489 and 0.9161 for MDD in GS:SFHS and UKB respectively, and 0.8173 and 0.8754 for AD in GS:SFHS and UKB respectively.

1 Dudbridge F. Power and predictive accuracy of polygenic risk scores. *PLoS Genet* 2013; **9**(3)**:** e1003348.

2 Palla L, Dudbridge F. A Fast Method that Uses Polygenic Scores to Estimate the Variance Explained by Genome-wide Marker Panels and the Proportion of Variants Affecting a Trait. *Am J Hum Genet* 2015; **97**(2)**:** 250-259.

3 Lubke GH, Hottenga JJ, Walters R, Laurin C, de Geus EJ, Willemsen G*, et al*. Estimating the genetic variance of major depressive disorder due to all single nucleotide polymorphisms. *Biol Psychiatry* 2012; **72**(8)**:** 707-709.

4 Ridge PG, Mukherjee S, Crane PK, Kauwe JS. Alzheimer's disease: analyzing the missing heritability. *PLoS One* 2013; **8**(11)**:** e79771.
